# Açaí Flan, A Functional Food with *Lacticaseibacillus rhamnosus* HN001 Probiotic: Physicochemical Characteristics, Probiotic Survival, Sensory Acceptance and Consumer Perception

**DOI:** 10.17113/ftb.62.01.24.8208

**Published:** 2024-03

**Authors:** Paulyne Tolentino Anselmo, Beatriz Cardoso Sabino, Carla Prado Rosolém, Márcia Simoni de Melo Rodrigues, José Renato Silva, Karla Bigetti Guergoletto, Tatiana Colombo Pimentel, Carina Moro Benis, Wilma Aparecida Spinosa, Giselle Nobre Costa

**Affiliations:** 1 Universidade Pitágoras Unopar, Programa de Mestrado em Ciência e Tecnologia de Leite e Derivados, Marselha street, 183, Zip code: 86041-140 Londrina, PR, Brazil; 2 Universidade Estadual de Londrina, Departamento de Ciência e Tecnologia de Alimentos, Celso Garcia Cid Road, PR 445, 86057-970 Londrina, PR, Brazil; 3 Instituto Federal do Paraná, Campus Paranavaí, Paranavaí, PR, Brazil

**Keywords:** *Lacticaseibacillus rhamnosus*, *Euterpe oleracea*, probiotic, dairy dessert, antioxidant, whey protein

## Abstract

**Research background:**

Açaí berry is rich in antioxidant compounds and is therefore closely associated with beneficial health effects. In this study, we aim to investigate the potential of using *Lacticaseibacillus rhamnosus* HN001 as a probiotic culture on açaí flan.

**Experimental approach:**

The chemical composition, physicochemical and microbiological characteristics, and sensory acceptance during refrigerated storage (5 °C for 42 days) of flan were investigated. In addition, the consumer perception of the product was evaluated using word association when consumers were shown a photo of the product with or without the added ingredients accompanied with a brief description of the product.

**Results and conclusions:**

The flan had a suitable chemical composition, mainly carbohydrates and proteins, probiotic viability reached 8 log CFU/g in the product and 4 log CFU/g after gastrointestinal simulation, typical açaí coloration, significant antioxidant activity and high sensory acceptability. The information about the ingredients and properties of the products increased the health value and positive feelings of the consumers towards the product.

**Novelty and scientific contribution:**

Açaí flan has proven to be a suitable carrier for *L. rhamnosus* HN001 as a probiotic culture, further enhancing the characteristic beneficial properties of the fruit. Therefore, combining this information with marketing strategies that inform consumers about the benefits of the product can further improve its acceptance. As far as we know, this is the first study on açaí flan with added probiotic culture.

## INTRODUCTION

Flan-type dairy desserts are considered popular foods around the world and have an important contribution to the diet due to their calcium and vitamin D contents, as well as phosphorus, potassium, magnesium, riboflavin, niacin, essential fatty acids and proteins (*1*). Açaí is a berry fruit native to Brazil and Bolivia whose properties have received a lot of attention in recent years, mainly due to the variety of phytochemicals such as polyphenols and its antioxidant activity, which are associated with many beneficial effects (*2**,**3*). In addition, açaí is a source of energy, fiber, anthocyanins, minerals and fatty acids, and has therefore been included in the top ten superfoods (*4*).

Probiotics are microorganisms that confer beneficial effects to individuals when consumed in adequate amounts. The *Lactobacillus rhamnosus* have recently been reclassified to *Lacticaseibacillus rhamnosus* (*5*). The HN001 strain has been associated with several health effects, such as modification of the intestinal microbiota, reduced prevalence of gestational diabetes and allergic diseases, among others (*6*,*7*).

Studies focusing on the addition of probiotic cultures to flans are still scarce (*8*-*10*) and, as far as the authors are aware, there is no application of the probiotic strain *L. rhamnosus* HN001 in flans and/or evaluation of the addition of probiotic cultures to berry açaí flans. Moreover, functional ingredients such as açaí and whey protein isolate are added to enrich the flan. This study therefore aims to evaluate the potential of the açaí flan as a carrier of the probiotic strain HN001. It also analyzed the consumer perception of the product and the impact of the available information.

## MATERIALS AND METHODS

### Lacticaseibacillus rhamnosus inoculum

Pasteurized milk (De Leite, Londrina, Brazil) was sterilized at 100 °C for 15 min, cooled and then 0.1 % (*m/V*) *L. rhamnosus* HN001™ (Dupont, Cotia, Brazil) was added. The mixture was incubated at 37 °C for 15 h (pre-inoculum). The pre-inoculum (*φ*=0.8 %) was added to the sterilized milk and incubated at 37 °C for 24 h. The pH, titratable acidity and viability of the probiotic were analyzed after 0, 3, 6, 9, 15, 18, 21 and 24 h of fermentation to determine the optimum fermentation time.

### Flan processing

The flan was prepared using the ingredientes: 23.6 % whole milk (3.4 % protein, 4.7 % carbohydrates and 3.5 % total fat; De Leite, Londrina, Brazil), 8 % whey protein isolate (Artesana, Novo Hamburgo, Brazil), 18 % sucrose (União, São Paulo, Brazil), 0.4 % citric acid (Anidrol, Lençóis Paulista, Brazil), 3 % açaí powder (Relva Verde, Ibiporã, Brazil), 35 % açaí pulp (Pura pulp, Guaraçaí, Brazil), 9 % probiotic culture inoculum and 3 % of unflavored gelatin (Green Grass, Ibiporã, Brazil).

The whole milk, whey protein isolate and sugar were weighed and heated in a water bath at 90 °C for 20 min. Then, the mixture was cooled in an ice bath to 40 °C, the other ingredients (citric acid, acai powder, acai pulp and unflavored gelatin) were added and homogenized in a mixer (Britânia, Curitiba, Brazil). The inoculum of the probiotic culture was then added and the mixture was packed in 30-mL plastic containers with a lid (Galvanotek, Carlos Barbosa, Brazil), in which the syrup had already been added. The flan syrup was made using 20 % açaí pulp, 0.3 % citric acid, 6 % water and 9 % sucrose. The mixture was heated until boiling for 5 min and then refrigerated at 5 °C. Analyses were performed weekly for 42 days.

### Viability of L. rhamnosus on flan and under simulated gastrointestinal conditions

The viability of *L. rhamnosus* HN001 was evaluated by plating on De Man, Rogosa and Sharpe (MRS) agar (Kasvi, São José dos Pinhais, Brazil) and anaerobic incubation at 37 °C for 72 h. The survival under simulated gastrointestinal conditions was monitored according to the method described by Minekus *et al*. (*11*), in which the food is subjected to different concentrations of enzymes and specific pH values at different times, aiming to simulate conditions in the mouth, stomach, small and large intestine, and the consequent survival of the probiotic in these conditions was measured *in vitro*.

### Physicochemical characteristics and antioxidant activity

The pH was evaluated using a pH meter (Kasvi, Curitiba, Brazil). Titratable acidity and chemical composition were determined according to AOAC SMPR® 2016.003 (*12*). The texture profile was determined using a TA3/1000 acrylic cylindrical probe (*d*=25.4 mm) and CT3 Texture Analyzer (Brookfield Ametek, Middleboro, MA, USA). Color parameters (L*, a* and b*) were determined using a colorimeter (CR-400; Konica Minolta, Ramsey, NJ, USA). The antioxidant activity was determined by the ability to sequester 1,1-diphenyl-2-picrylhydrazyl (DPPH) radical and expressed as EC_50_/(mg/mL) according to the method described by Brand-Williams *et al*. (*13*), and using the iron reduction method (FRAP) expressed as Fe^2+^/(mmol/g) according to Benzie and Strain (*14*). The reagents used were of analytical grade (Sigma-Aldrich, Merck, São Paulo, Brazil).

### Sensory analysis

The acceptance (overall impression, appearance, color, aroma, flavor and texture) of the flan (30 g) was evaluated by 100 consumers of which 51 men and 49 women, recruited by direct approach and invitation for sensory evaluation. This sensory test uses a 9-point structured hedonic scale (1=very much disliked, 9=very much liked) and the purchase intent was assessed using a 5-point scale (1=would definitely buy, 5=would definitely not buy).

### Consumer perception evaluation

**Fig. S1:**
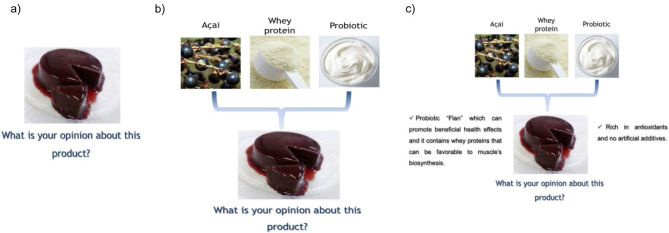
The images were sequentially (a, b, c) shown to the consumers to evaluate the perception of the product

The participants (*N*=474) received *via* e-mail or social media three different images (Fig. S1) and the attributed characteristics. The words provided as answers were analyzed. They were divided into three groups (*N*=158): group 1 (received a photo of the product, Fig. S1a), group 2 (received a photo of the product providing the ingredients used, Fig. S1b), and group 3 (received a photo of the product providing the ingredients used and a short description of the product, Fig. S1c). All the consumers were asked to describe their opinion of the product. The words, descriptions and associations provided by the participants were considered for the analysis as described by Pinto *et al*. (*15*).

### Statistical analysis

The experiment followed a completely randomized design and was repeated three times. Physicochemical and microbiological analyses were performed in triplicate. Data were submitted to analysis of variance (ANOVA) followed by the Tukey’s test (p<0.05) using STATISTICA software, v. 8.0 (*16*). The data of word association were analyzed according to Pinto *et al*. (*15*).

## RESULTS AND DISCUSSION

When selecting a probiotic microorganism to be included in a food product, the matrix in which it will develop plays a fundamental role in its multiplication and maintenance throughout its shelf life. Therefore, defining the growth medium can be crucial for the functionality of a probiotic strain. The incubation of milk caused a decrease in pH from 6.6 to 4.9 and an increase in titratable acidity from 0.2 to 0.5 % lactic acid over 24 h (Table 1). The decrease in pH and the increase in acidity are the result of the fermentation of the milk by the probiotic culture, which uses lactose and other sugars present in the medium and produces lactic acid (*17*). The viability of the probiotic culture decreased after 3 h of fermentation (from 8.3 to 7.9 log CFU/g), increasing during the incubation period and reaching 8.9 log CFU/g after 24 h (p<0.05). The initial decrease is due to the period of adaptation of the culture to the environment. There was no significant difference in the number of probiotic culture between 15 and 24 h of fermentation (p>0.05). Therefore, considering the time and energy savings, it is suggested to use 15 h of fermentation to obtain the inoculum. Here, 24 h was used to facilitate logistics. Studies that used *L. rhamnosus* under similar conditions reported viability of 8 and 9 log CFU/g for the used strains and maintenance of these values until the end of the shelf life (*18**,**19*).

**Table 1:** Viability of the probiotic culture and physicochemical characteristics (pH and titratable acidity) of milk during 24 h of fermentation

*t*/h	*N*/(log CFU/g)	pH	Titratable acidity/%
0	(8.3±0.3)^c^	(6.6±0.0)^a^	(0.2±0.0)^de^
3	(7.9±0.1)^d^	(6.1±0.2)^c^	(0.21±0.05)^e^
6	(8.4±0.1.5)^c^	(6.42±0.05)^ab^	(0.22±0.01)^e^
9	(8.52±0.11)^bc^	(6.34±0.05)^b^	(0.22±0.01)^de^
15	(8.72±0.02)^abc^	(5.70±0.10)^d^	(0.30±0.01)^cd^
18	(8.91±0.01)^a^	(5.41±0.05)^e^	(0.43±0.05)^bc^
21	(8.81±0.10)^ab^	(4.90±0.05)^f^	(0.53±0.05)^ab^
24	(8.90±0.04)^ab^	(4.90±0.05)^f^	(0.50±0.05)^a^

Results are expressed as mean value±standard deviation (*N*=9). Different lowercase letters in the same column denote significant difference by the Tukey’s test (p<0.05). Titratable acidity is expressed as lactic acid

The açaí flan consisted of (g/100 g): moisture 58.6, protein 15.5, lipid 0.3, ash 0.8 and carbohydrates 24. Therefore, it is characterized as a dairy product with high protein and carbohydrate contents and low lipid content.

The açaí flan had pH=4.6 and titratable acidity of 0.1 % lactic acid (Table 2). During storage, the pH of the products decreased from 4.64 to 4.57 and the titratable acidity increased from 0.09 to 0.1 % lactic acid. The decrease in pH and the increase in acidity are a result of post-acidification of the products promoted by probiotic culture, which used lactose and other sugars present in the medium and produced lactic acid (*17*). The acidification of the product was mild (0.07 pH units and 0.01 % lactic acid), demonstrating that the probiotic culture does not have high fermentative capacity at low temperatures (*18**,**19*), which is interesting from the sensorial point of view as well as the stability during the shelf life.

**Table 2:** Physicochemical characteristics and color parameters of açaí flan during refrigerated storage at 5 °C

*t*(storage)/day	pH	Titratable acidity/%	L*	a*	b*	Hardness/N	Adhesiveness/mJ	Cohesiveness	Gumminess/N	EC_50_/(mg/mL)	FRAP as *b*(Fe^2+^)/(mmol/g)
1	(4.64±0.07)^ab^	(0.09±0.01)^b^	(39.6±3.9)^a^	(10.9±1.6)^a^	(5.9±1.6)^b^	(4.5±0.7)^b^	(5.8±1.0)ª	(0.39±0.03)^b^	(2.0±0.5)^b^	(111.3±2.5)^d^	(1.43±0.01)^a^
7	(4.7±0.1)^a^	(0.09±0.01)^b^	(37.2±3.1)^a^	(4.1±1.9)^b^	(4.6±1.8)^b^	(5.3±0.6)^ab^	(5.5±0.4)ª	(0.4±0.4)^b^	(2.1±0.6)^b^	(165.7±9.9)^c^	(1.37±0.02)^a^
14	(4.57±0.06)^abc^	(0.09±0.00)^b^	(33.4±2.5)^b^	(6.5±0.5)^b^	(8.3±0.7)^a^	(4.6±0.6)^ab^	(5.1±0.8)^a^	(0.37±0.03)^b^	(1.9±0.7)^b^	(170.2±5.8)^bc^	(1.33±0.02)^a^
21	(4.46±0.05)^cd^	(0.09±0.01)^b^	(34.3±2.7))^b^	(7.3±0.7)^b^	(7.0±1.3)^ab^	(6.2±0.8)^ab^	(5.4±1.0)^a^	(0.39±0.03)^b^	(3.0±0.6)^ab^	(184.8±3.5)^bc^	(1.31±0.01)^a^
28	(4.3±0.1)^d^	(0.11±0.01)^a^	(33.3±2.6)^b^	(7.2±0.9)^b^	(7.4±0.8)^ab^	(5.2±0.9)^ab^	(5.1±0.7)^a^	(0.38±0.07)^b^	(2.2±1.0)^b^	(193.0±7.5)^b^	(0.84±0.02)^b^
35	(4.4±0.2)^cd^	(0.11?±0.01)^a^	(33.7±2.8)^b^	(6.9±1.4)^b^	(8.2±1.4)^ab^	(6.3±2.3)ª	(1.2±0.9)^b^	(0.54±0.08)ª	(4.6±1.1)ª	(426.1±8.31^a^	(0.74±0.01)^b^
42	(4.6±0.1)^bc^	(0.11±0.01)^a^	(36.9±3.0)^a^	(6.4±3.0)^b^	(8.1±1.7)^ab^	(5.8±1.4)^ab^	(0.3±0.3)^b^	(0.6±0.4)ª	(4.2±1.0)ª	(445.5±6.7)^a^	(0.81±0.02)^b^

Results are expressed as mean value±standard deviation (*N*=9). Different lowercase letters in the same column denote significant difference according to Tukey’s test (p<0.05). Titratable acidity is expressed as lactic acid

The açaí flan had a red-purple color (L*=39.6, a*=10.9 and b*=5.87) (Table 2), which is typical for açaí pulp. During storage, there was a decrease in the red color (lower values ​​of a*, p<0.05) and maintenance of the parameters L* and b* (p>0.05) when comparing the products on day 1 and 42 of storage. Red discoloration of the products during storage could be related to a decrease in the anthocyanin concentration.

The flan was characterized as a soft and adhesive product, with a hardness of 4.54 N, adhesiveness of 5.8 mJ, cohesiveness of 0.39 and gumminess of 1.99 N (Table 2). During storage, there was a decrease in the adhesiveness and an increase in the cohesiveness and gumminess (p<0.05). In addition, there was an increase in the firmness for up to 35 days of storage with subsequent decrease, without significant difference between the freshly prepared product (day 1) and that stored for 42 days (p>0.05). The texture parameters are characteristic of protein gels. The addition of whey protein concentrate improves gelatinization with protein-protein interactions. Frederico *et al*. (*9*) and Costa *et al*. (*19*) also observed similar properties of flans or ice cream with added whey. The acidification of the product observed during refrigerated storage may have contributed to the increase in firmness, cohesiveness and gumminess of the product, as well as to the decrease of adhesiveness.

The flan had an antioxidant activity of EC_50_=111.34–445.50 mg/mL (DPPH method) and *b*(Fe^2+^)=0.81–1.43 mmol/g (FRAP, Table 2), which can be considered appropriate from a health point of view. During the storage period, in the antioxidant activity decreased (higher values for DPPH and lower values for FRAP, p<0.05), which may be related to the loss of anthocyanins.

The açaí flan had probiotic culture counts of 8.43–8.60 log CFU/g during the 42 days of refrigerated storage (Table 3). The minimum number of viable probiotic cells in a product should be in the range of 6–7 log CFU/g to observe the beneficial effects (*10*). Thus, the flan prepared in the present study can be considered as a probiotic product throughout the storage period.

**Table 3:** Viability of probiotic culture in the product and under simulated gastrointestinal conditions

*t*(storage)/ day	*N*(*Lacticaseibacillus rhamnosus* HN001)/(log CFU/g)			
Product	Gastric phase	Small intestine	Large intestine
1	(8.4±0.4)^aA^	(6.11±0.01)^bA^	(3.2±0.2)^dA^	(4.3±0.2)^cA^
14	(8.6±0.3)^aA^	(4.9±0.3)^bA^	(3.2±0.5)^cA^	(4.7±0.2)^bA^
28	(8.5±0.3)^aA^	(5.3±0.2)^bA^	(3.6±0.3)^cA^	(417±0.2)^bA^
42	(8.6±0.2)^aA^	(5.57±0.07)^bA^	(3.15±0.01)^dA^	(4.58±0.08)^cA^

Different lowercase letters in superscript in the same row indicate a significant difference on the same day at the different stages of digestion (p<0.05). Different capital letters in superscript in the same column indicate significant differences in each phase of digestion in the gastrointestinal tract during refrigerated storage (p<0.05)

The açaí flan had probiotic culture counts of 4.91–6.11, 3.17–3.60 and 4.07–4.58 log CFU/g during the gastric and enteric (small and large intestine) phases, respectively. Thus, the probiotic culture *L. rhamnosus* was able to survive the simulated gastrointestinal conditions. There was a 3 log CFU/g decrease during the gastric phase, with subsequent recovery of probiotic culture in the enteric phases (~1 log CFU/g, p<0.05). It is possible that the probiotic was only damaged during the gastric phase, with consequent decrease in its counts, but when subjected to favorable conditions for its survival, the microorganism recovered viability, showing higher counts in the first and second enteric phases. Costa *et al*. (*19*) reported a 5-log cycle reduction of the *L. rhamnosus* GG in açaí ice cream when subjected to gastrointestinal tract simulation.

The flan received scores above 7 on a 9-point hedonic scale for aroma, flavor and texture, indicating that consumers moderately liked these attributes in the product. In addition, the flan received scores above 8 for the attributes of appearance, color and overall impression, indicating that consumers liked these attributes very much. Product acceptance was 89 %. Regarding purchase intention, 60 % of consumers said that they would certainly or probably buy the product and only 4 % said they would definitely not buy it.

Related to the consumer perception of the product, the consumers received three different images (Fig. S1) and attributed characteristics. The words they used to describe them were considered for the analysis.

To analyze consumer perceptions, the researchers sent invitations through social media for voluntary participation in the survey. The participants were shown a sequence of product images over three different weeks (Fig. S1a), an image together with the ingredients (Fig. S1b), and an image with some attributed beneficial effects (Fig. S1c). They were asked to express their opinion of the product using a single word and the responses received were grouped to create an analysis map. The words associated with the product were categorized as follows: composition, health, positive feelings, negative feelings, sensory perception and description (Table 4). It was observed that the inclusion of the information on the ingredients used and, especially the description of the product (Fig. S1c) led to an increase in the perception of health and positive feelings among the consumers. In fact, clinical trials (*20*) have shown that the consumption of açaí has many beneficial effects on health. Due to its medicinal properties and the absence of undesirable effects, açaí and foods containing it have a promising future and great economic potential in the food and cosmetic industry.

**Table 4:** Contingency table showing the main sensory descriptors and the frequency of use of each by consumers

Attribute	Example	Control	Ingredient	Ingredient and description
Composition	Caloric, fat, sugar, protein, anthocyanins, phenolic compounds, bioactive compounds, vitamins, lactose, fiber, flavonoids, nutrients	9(-)**	52(+)**	36
Health	Antioxidant, healthy, nutritious, energetic, functional, probiotic, microbiota regulation, strength, well-being, diet, fitness, supplementation, beneficial microorganisms, metabolism, muscle, satiety, digestive, enriched, young, intestine	1(-)***	145(+)**	165(+)***
Positive feelings	Delicious, beautiful, pleasure, tasty, attractive, good, colorful, showy, appetizing, different, refreshing, fresh	61	94(-)*	130(+)*
Negative feelings	Strange, too industrialized, bad, unhealthy, fattening, cracked, not appetizing, anxiety, blood, artificial, sickening, too sweet, bitter, bland taste, residual taste, sandiness	18	23	20
Sensory perception	Icy, sweet, soft, red fruit flavor, bright, chocolate, creamy, moist, juicy, firm, good appearance, beautiful color, flavor, texture, acid, homogeneous, strawberry	117(+)***	79(-)*	58(-)***
Description	Pudding-like, flan, with syrup, guava-paste like, gelatin-like, sweet cheese-like, açaí-like, semi-solid, dark, natural, chocolate cake-like, ice cream like	24(+)*	29	24

The number of citations for each sensory test and the results of the chi-squared analysis per cell are shown. (+) or (−) indicate that the observed values are higher or lower than the expected theoretical value. Categories mentioned by at least 5 % of respondents. *p<0.05, **p<0.01, ***p<0.001

## CONCLUSIONS

Açaí flan can be a carrier of *Lacticaseibacillus rhamnosus* HN001 as a probiotic culture that has a suitable chemical composition, physicochemical properties and antioxidant activity as well as sufficient probiotic culture counts in the product and under simulated gastrointestinal conditions. The methodology used to evaluate consumer perceptions, which included voluntary participation through social media, enabled a clear association of flan images and ingredients with aspects of health and positive feelings among consumers. The findings contribute to the evolving functional food landscape by combining scientific research with consumer perception analysis to clarify the potential impact of probiotic and açaí-based products.

## Figures and Tables

**Table:**
